# Influence of synthesis parameters on CCVD growth of vertically aligned carbon nanotubes over aluminum substrate

**DOI:** 10.1038/s41598-017-10055-0

**Published:** 2017-08-25

**Authors:** Anna Szabó, Egon Kecsenovity, Zsuzsanna Pápa, Tamás Gyulavári, Krisztián Németh, Endre Horvath, Klara Hernadi

**Affiliations:** 1 Department of Applied and Environmental Chemistry, University of Szeged, Szeged, 6720 Rerrich B. ter 1, Hungary; 2 MTA-SZTE “Lendület” Photoelectrochemistry Research Group, University of Szeged, Szeged, 6720 Rerrich B. ter 1, Hungary; 3 Department of Optics and Quantum Electronics, University of Szeged, Szeged, 6720 Dom ter 9, Hungary; 4 Laboratory of Physics of Complex Matter (LPMC), Ecole Polytechnique Federale de Lausanne, Centre Est, Station 3, CH-1015 Lausanne, Switzerland

## Abstract

In the past two decades, important results have been achieved in the field of carbon nanotube (CNT) research, which revealed that carbon nanotubes have extremely good electrical and mechanical properties The range of applications widens more, if CNTs form a forest-like, vertically aligned structure (VACNT) Although, VACNT-conductive substrate structure could be very advantageous for various applications, to produce proper system without barrier films i.e. with good electrical contact is still a challenge. The aim of the current work is to develop a cheap and easy method for growing carbon nanotubes forests on conductive substrate with the CCVD (Catalytic Chemical Vapor Deposition) technique at 640 °C. The applied catalyst contained Fe and Co and was deposited via dip coating onto an aluminum substrate. In order to control the height of CNT forest several parameters were varied during the both catalyst layer fabrication (e.g. ink concentration, ink composition, dipping speed) and the CCVD synthesis (e.g. gas feeds, reaction time). As-prepared CNT forests were investigated with various methods such as scanning electron microscopy, Raman spectroscopy, and cyclic voltammetry. With such an easy process it was possible to tune both the height and the quality of carbon nanotube forests.

## Introduction

Vertically aligned carbon nanotubes (VACNTs) henceforth denoted also as carbon nanotube forests (CNT forest) were first synthesized in 1996^1^, and subsequently they got into the focus of research in nanotechnology. Catalytic chemical vapor deposition (CCVD) proved to be the most efficient way for the production of vertically aligned CNTs. The most common and efficient catalysts are the mono- or bimetallic transition metals (Fe, Co, Ni), while Al_2_O_3_, SiO_2_ or MgO are generally applied as supports^2–6^. Breakthrough in development occurred in 2004 when Hata *et al*.^7^ modified the CNT growth and introduced a small amount of water into the CVD chamber together with the carbon source. Lot of studies have been published so far related successful CNT growth^8^, however, the properties of both the support and the catalytic layer considerably affect the properties of CNT forests, i. e. density, orientation, length, thickness and graphitization of the product, and therefore further investigations are necessaryin this field.

In 2001, it was shown by Mauron *et al*. that carbon nanotubes can be oriented perpendicularly to the substrate surface with high densities of nucleation centers, moreover, high CNT density can be achieved by using high quantity of iron oxide clusters which facilitated the growth in the desired orientation^9^.

The CVD parameters (such as carbon source, gas feed, reaction time, reaction temperature, etc.) also have significant role in the formation of VACNTs. Iijima *et al*. have investigated the kinetics of water-assisted CVD by a quantitative time-evolution analysis and concluded that the complex behavior of the time evolution of supergrowth can be easily explained by analyzing the two fitting parameters of the simple growth model, i.e. initial growth rate and the characteristic catalyst lifetime^10^. Since then only a few papers were published which attempted to find correlation between gas feed (mostly acetylene as carbon source, argon as carrier and hydrogen as reducing agent) and the characteristics of vertically aligned carbon nanotubes^11–14^.

From the perspective of the catalyst, the thickness, composition, density and adherence of the transition metal layer are crucial parameters and have major effect on the properties of CNT growth. The applied techniques so far are very effective but rather expensive such as magnetron sputtering^15, 16^, radio frequency sputtering^17, 18^, electron beam evaporation^19–21^ and physical vapor deposition^22, 23^, therefore developing a cheap, easy-to-handle method would be desirable. In 2003 it was already pointed out that high-quality but not aligned SWNTs can be synthesized directly onto silicon and quartz substrates using the easy and costless dip-coating approach for the deposition of catalytic metals^24^.

Nyikos *et al*. reported that by using an efficient dip coating process, a metal substrate and an optimized Fe:Mo (47:3) catalyst system, the CNT diameter, specific surface area and gravimetric capacity of the forest could be controlled^25^. Vertically aligned CNTs were reproducibly synthesized also onto a metal surface (bulk Cu) by Shanov *et al*. and the effects of Ti, Ni, and Ni–Cr intermediate layers were found to play an important role in achieving vertical alignment of CNTs^26^.

While the results about precise growth mechanism of carbon nanotubes are still rather contradictory, there are a couple of points during CVD growth wherein researchers agree^27–32^, namely, i) the process starts with the formation and then the reduction of the nanoparticles from the initially homogeneous catalyst layer (even in the absence of carbon source) ii) and continues with the nucleation and the growth of carbon nanotubes. The importance of a (native) oxide layer on the substrates is also well known since it promotes the transformation of metal films, such as Fe, Co, Ni and their alloys, into catalyst insulated nanoparticles^33, 34^ which can facilitate the growth of CNTs. Although, VACNT-conductive substrate structure could be very advantageous for various applications, to produce proper system without barrier films i.e. with good electrical contact is still a challenge.

The aim of this work was to investigate the controlled growth of vertically aligned carbon nanotubes over a simple aluminum plate. The effect of synthesis parameters was followed by scanning and transmission electron microscopies as well as Raman spectroscopy.

## Experimental

### Materials

As for substrate aluminum plate produced by WRS Materials Company was used in every experiment. For building catalyst layer cobalt (cobalt(II)-nitrate hexahydrate, 99% (Sigma-Aldrich)) and iron (iron(III)-nitrate nonahydrate, 99.9% (Sigma-Aldrich)) precursors were dissolved in absolute ethanol (VWR). During the CVD synthesis the gas feed contained ethylene, hydrogen and nitrogen (all supplied by Messer Hungary).

### Catalyst preparation

First the ethanolic solution of catalyst metals was prepared using *Fe*(*NO*_3_)_3_ × 9*H*_2_ *O* and *Co*(*NO*_3_)_2_ × 6*H*_2_ *O* salts. The ratios of catalyst metals varied was as follows: Fe:Co = 0:1, 1:3, 2:3, 1:1, 3:2, 3:1, and 1:0, respectively. The concentrations of the ink (iron and cobalt containing ethanolic solution) were 0.022 M, 0.044 M, 0.066 M, 0.11 M, 0.22 M, 0.44 M and 0.66 M, respectively. In most cases the following catalyst solution was used: 0.11 M with the Fe:Co ratio of 2:3 for which 0.888 g *Fe*(*NO*_3_)_3_ × 9*H*_2_ *O* and 0.855 g *Co*(*NO*_3_)_2_ × 6*H*_2_ *O* were homogenized in ethanol in a volumetric flask of 50 cm^3^. In order to prevent the effect of aging which might modify the properties of the solution^35^ catalyst inks were always prepared freshly before dip coating.

In the second step the support (aluminum sheet) was pretreated before catalyst deposition. In order to remove any contamination (motes, grease spots, etc.) from its surface the aluminum sheet was placed into an ultrasound bath containing distilled water then it was washed with ethanol and acetone sequentially. After that the decontaminated support was heat treated at 400 °C in a static oven for 1 h which resulted in a native oxide layer of higher thickness. This alumina layer is fairly advantageous for two reasons, it serves an effective catalyst support in CVD, and also provides better wetting of the surface by the ink. For dip coating and CVD 3 × 2.5 cm sized support sheets were prepared.

Dip coating is a flexible method to build thin layers onto the surface of catalyst support under controlled conditions. Before dip coating aluminum sheets were flattened for better equipartition. For the catalyst preparation a KSV dip coater LM (KSV Instruments Ltd.) was used. Dipping speed was set to 200 *mm* × *min* ^−1^ in every experiment, and the speed of withdrawal varied between 50–200 *mm* × *min* ^−1^. Aluminum plates were kept in the ink for 5 seconds. In order to stabilize the metal nitrate layer in oxide state the dip coated samples were heated again at 400 °C (with a heating rate of 30 °C × *min* ^−1^) in a static oven for 1 h.

### CCVD synthesis

For the synthesis of CNT forests the common CCVD method was applied. Before synthesis, the size of the catalyst samples on aluminum plate were further reduced to 3 × 0.4 cm to fit the quartz boat since the diameter of quartz tube was 20 mm. As the melting point of aluminum is 660 °C all CCVD syntheses were carried out at 640 °C. The gas feed contained ethylene as carbon source (70–120 *cm* ^3^ × *min* ^−1^), nitrogen as carrier (50–75 *cm* ^3^ × *min* ^−1^), hydrogen as reductive agent (100–130 *cm* ^3^ × *min* ^−1^), and – in certain experiments – water vapor (32–42 *cm* ^3^ × *min* ^−1^).

**Figure 1:**
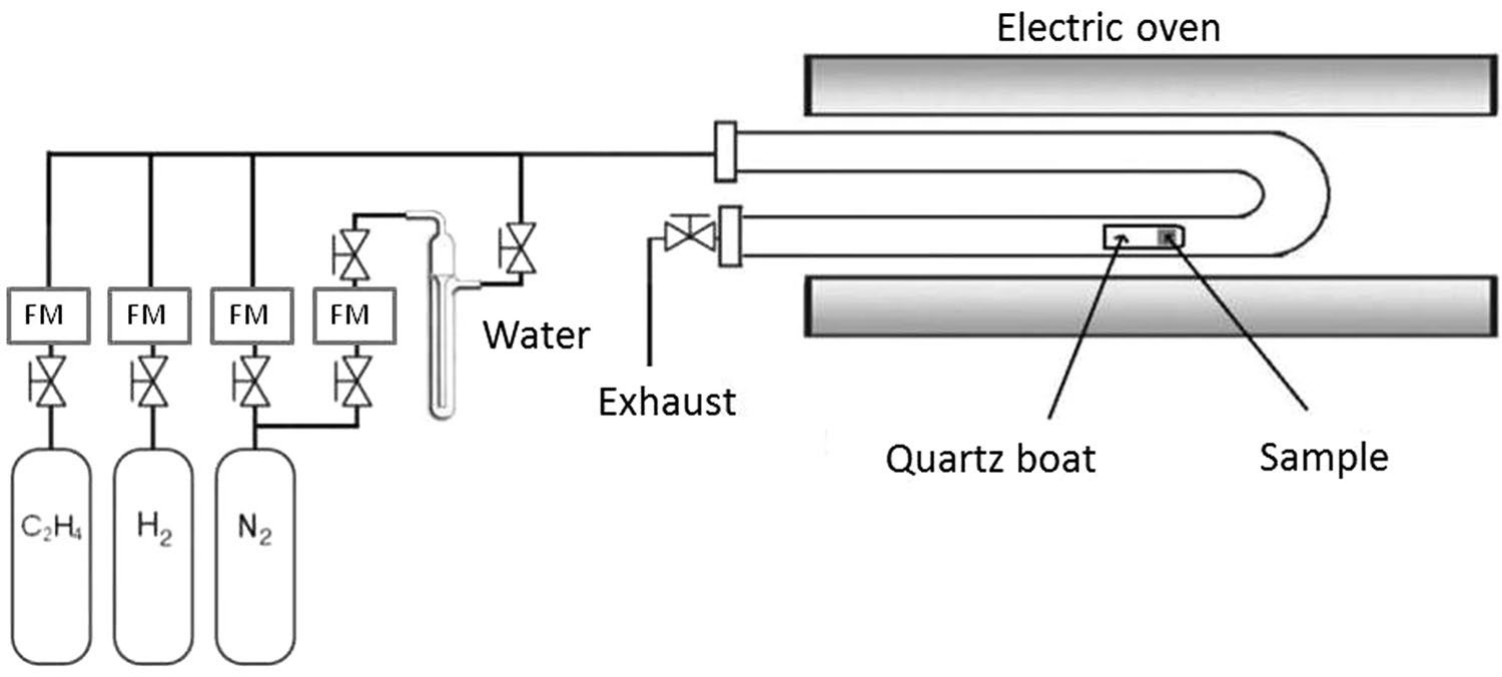
Schematic image of CCVD reactor.

The schematics of the CCVD reactor is shown in Fig. 1. In a typical synthesis first the reactor was rinsed by nitrogen to remove oxygen traces from the system. Then the catalyst sample in a quartz boat was placed into the reactor tube under continuous nitrogen flow. When the system reached the reaction temperature hydrogen was added to the gas feed for 5 mins in order to reduce the catalyst sample. The CCVD reaction was initiated with the addition of ethylene (and water vapor) flow. Reaction time varied from 2 to 30 mins, however, most frequently 15 min run was applied. After the synthesis all gas feed turned off except nitrogen. Then the system was rinsed for 2 min after that the quartz tube was removed from the oven and was cooled down to room temperature under continuous nitrogen flow.

### Characterization of CNT samples

To investigate the morphology of CNT forests Scanning Electron Microscopy (SEM) analysis was carried out with a Hitachi S-4700 Type II FE-SEM (5–15 keV) instrument. In order to take images of the forests from side, the sample holder was turned at an angle of 35^◦^ during the measurements. Heights of the forests were calculated regarding this value. SEM results were evaluated by using ImageJ software.

The quality of carbon nanotubes was examined via (high resolution) Transmission Electron Microscopy (TEM) using a Philips CM 10 (100 keV) type instrument. During sample preparation CNT bunches were collected from the surface of Al substrate which were suspended in 1.25 cm^3^ absolute ethanol. From this suspension 2–3 droplets were deposited onto the surface of a holey carbon grid (Lacey, CF 200). Images were analyzed by using Soft Imaging Viewer software.

The quality and graphitic properties of carbon nanotube forests were investigated by Raman spectroscopy. The measurements were carried out with Thermo Scientific DXR Raman microscope with 532 nm excitation wavelength.

## Results and Discussion

### Catalyst preparation

Since the properties of catalyst layer are crucial regarding the CNT forest growth during CCVD synthesis, parameters affecting catalyst deposition were examined first. The effect of dip coating speed and the concentration/ratio of catalyst ink on the features of CNT forest growth were investigated in detail.

#### The effect of dip coating

In this series the speed of withdrawal varied in the range of 50–200 *mm* × *min* ^−1^ but the concentration and the metal ratio of catalyst ink were fixed at values of 0.11 M and 2:3, respectively. CCVD parameters were the following: the flow rates for ethylene, hydrogen, nitrogen and water vapor were 70, 100, 50 and 30 *cm* ^3^ × *min* ^−1^, respectively, while the reaction time was 15 minutes in each run. Our former result revealed that the influence of aging in case of ethanolic solution of *Fe*(*NO*_3_)_3_ × 9*H*_2_ *O* is a significant issue. The color changes to dark reddish brown in a few hours which is also an important sign of oligomer formations^35^^.^ This phenomenon is disadvantageous during dip coating therefore always freshly prepared ink was used for layer preparation. From the SEM analysis of grown CNT forests a qualitative conclusion was drawn. Both the height and quality of CNT forest are rather poor when the lowest dip coating speed was applied. A tendency was observed that increasing the speed of withdrawal results in higher forests with progressively better orientation.

#### The effect of catalyst ratio

**Figure 2:**
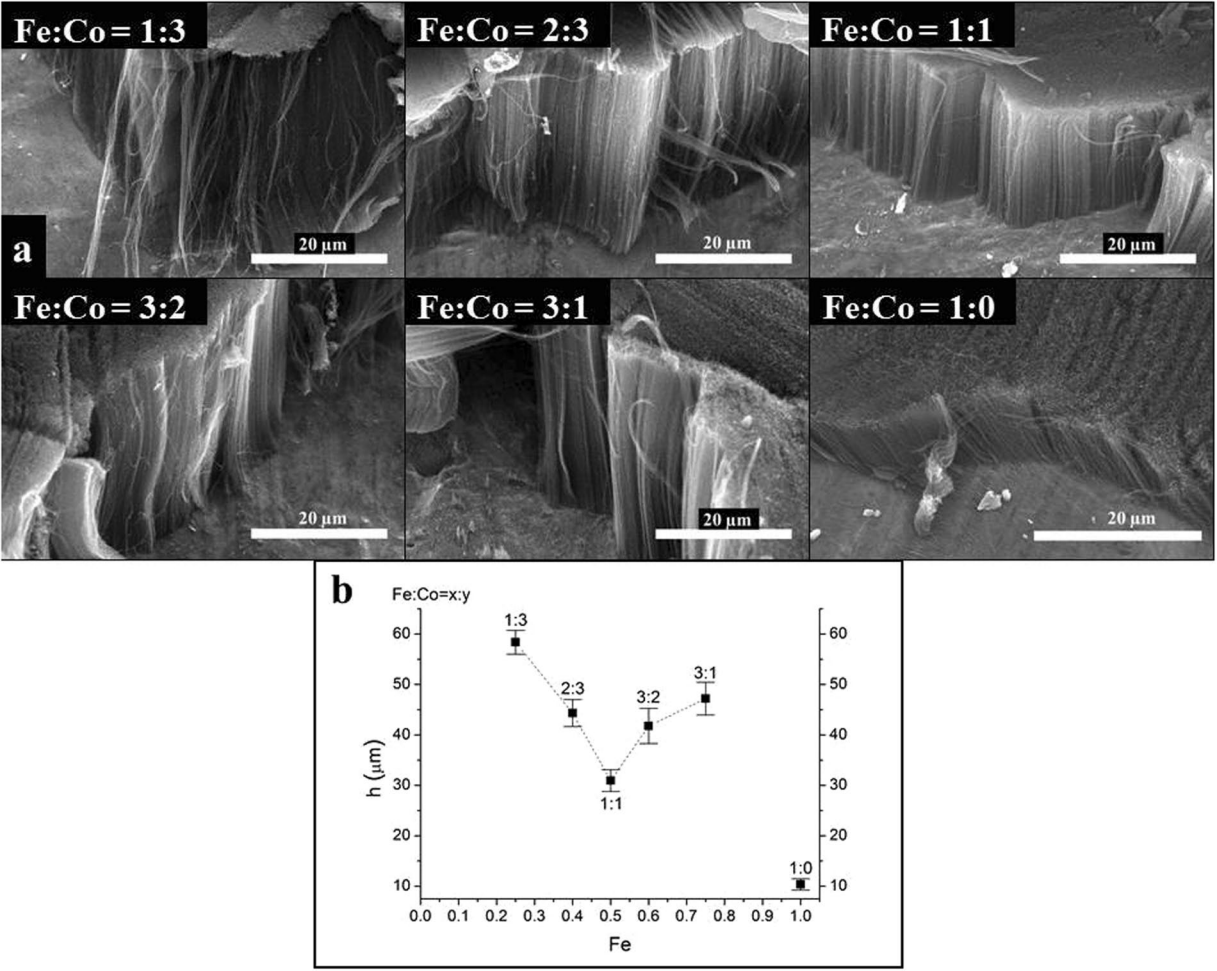
SEM images of CNT forests synthetized at various catalyst ratios (**a**) and heights of CNT forests (**b**).

In these experiments the effect of Fe:Co ratio of catalyst ink on the properties of CNT forests was investigated. While CCVD parameters and ink concentration were the same as mentioned in the previous paragraph, the ratio of two catalyst metals varied as follows: Fe:Co = 0:1, 1:3, 2:3, 1:1, 3:2, 3:1, and 1:0, respectively. Monometallic catalyst layers are of great importance since both iron and cobalt were reported to be active in normal CCVD carbon nanotube synthesis, moreover Fe alone proved to be suitable for growing CNT forest^36^. However, in our system no carbon deposit could be observed in case of using pure metal salt as ink. It can be concluded that for CNT forest synthesis bimetallic catalyst layer is required on the surface of Al support. It is known from the literature that the role of alumina is advantageous since suppresses the diffusion and aggregation of catalyst nanoparticles by forming metal-dissolved alumina after catalyst metals diffuse into alumina and precipitate as nanoparticles^37^. In our case native oxide layer was developed by heat treatment in order to avoid dissolution of catalysts in metallic substrate. Alumina is considered as a key material to initiate catalysis of carbon nanotube growth. The effect of Fe:Co ratio on CNT forest was investigated via SEM (Fig. 2a).

It was revealed that all bimetallic layers were active in CCVD synthesis. Although in the literature other research groups used the 1:1 ratio most often in our system this 1:1 ratio resulted in the lowest carbon nanotube forest. SEM images pointed that there is only a slight difference in the quality and orientation of CNT forests, presumably only a slight preference can be concluded for samples of higher cobalt content. Plotting the height of CNT forest as a function of catalyst composition (see Fig. 2b) it was found that Fe:Co ratio has a strong influence on the growth. While the lowest value, approx. 20 µm was obtained when the Fe:Co ratio was 1:1, the highest (almost 3 times higher) CNT forest of almost 60 µm grew on the layer in case of 1:3 ratio. In spite of the fact that Magrez *et al*. have already proved the positive role of Fe_2_Co^38^, catalyst layers of other composition resulted in CNT forests of 40–50 µm height.

### The effect of ink concentration

**Figure 3:**
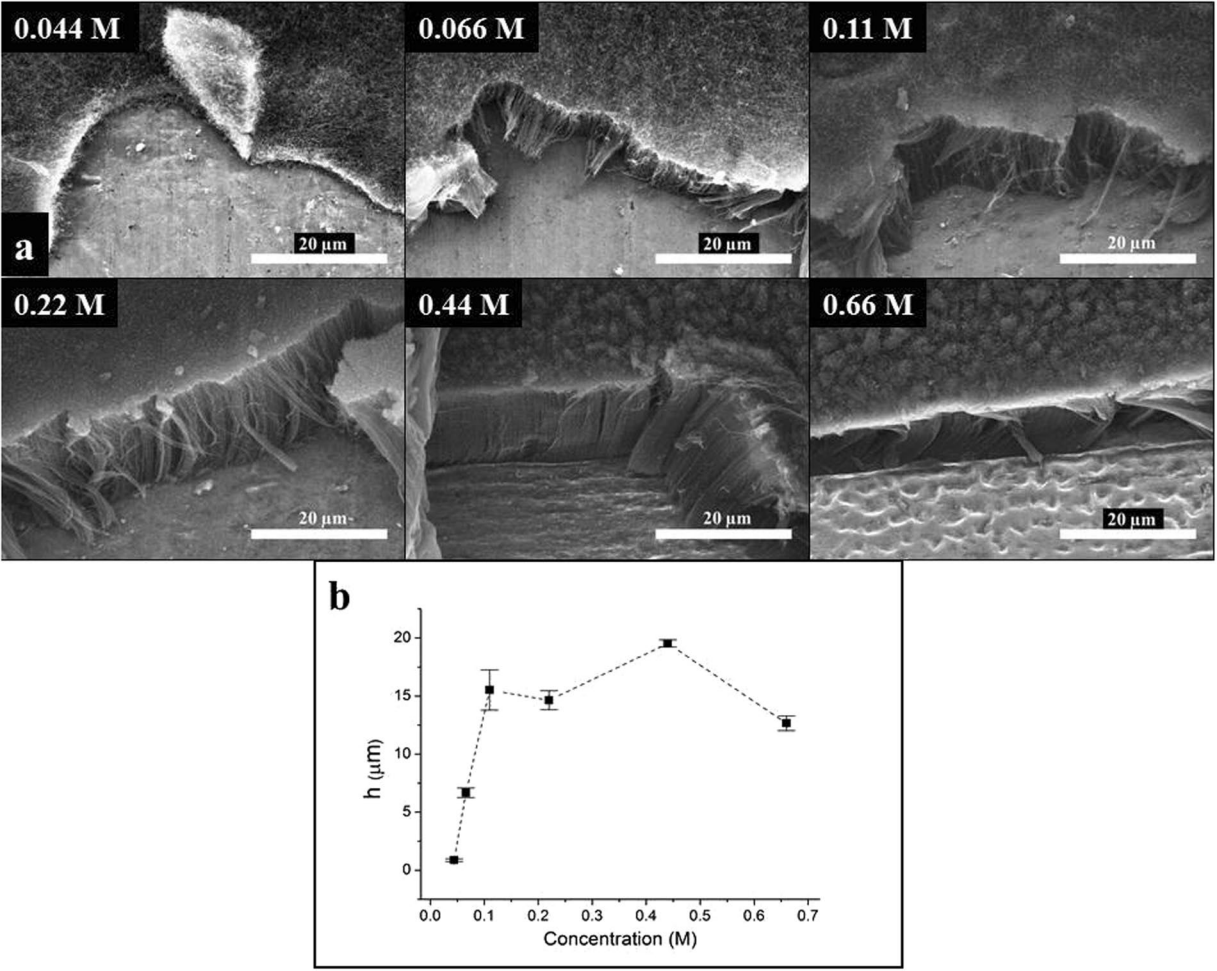
SEM images of CNT forests synthesized by varying the catalyst ink concentration (**a**) and heights of CNT forests (**b**).

Thereinafter the effect of ink concentration on CCVD growth was studied. While CCVD parameters and Fe:Co ratio were the same as mentioned before, the concentrations of the catalyst ink varied as follows: 0.022 M, 0.044 M, 0.066 M, 0.11 M, 0.22 M, 0.44 M and 0.66 M, respectively. During the investigation of CNT forests grown over layers using various catalyst ink concentrations, it was demonstrated, that ink with the lowest concentration cannot be used for layer formation since no significant carbon deposit was observed on the surface of Al substrate. As it can be seen in Fig. 3a, applying catalyst ink of 0.044 M carbonaceous material has deposited onto the surface during CCVD synthesis, nevertheless, only disordered carbon nanotubes can be observed. By applying higher ink concentrations well-aligned carbon nanotubes were formed, however, even from SEM analysis it can be seen that both their orientation and density changed with concentration (Fig. 3a). Diagram in Fig. 3b illustrates the height of CNT forests as a function of ink concentration.

It can be deduced that ink concentrations lower than 0.11 M result in proportionally lower CNT forest, nevertheless this tendency does not continue above 0.11 M. Instead, the heights of CNT forests varied in the range of 12 − 18 µm, consequently increasing ink concentration has no significant influence on the height. According to our results in the range of low ink concentrations, the carbon nanotube forests’ height is affected dramatically by the ink concentration, the height varied between 0.87−6.66 µm. The growth of the highest carbon nanotube forests can be observed in the case of the reference ink concentration (0.11 M), meaning it is the optimum concentration for the synthesis of carbon nanotube forests with relatively increased height. In the section “Further analysis of carbon nanotubes composing forests” it is discussed that the diameter of the carbon nanotubes is not influenced by the ink concentration, supported also by the TEM images.

**Figure 4:**
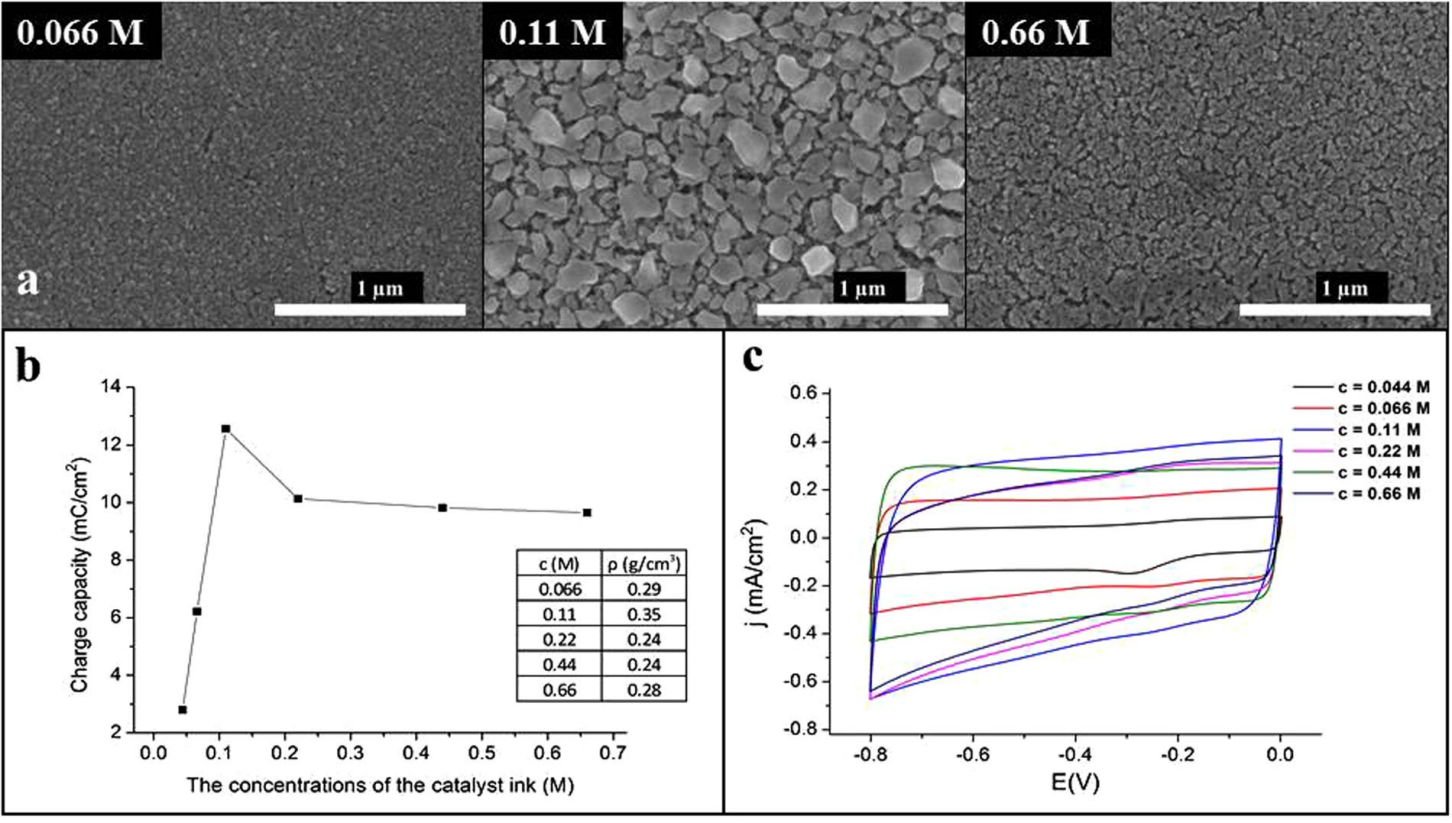
SEM images of CNT forests pre-synthesized (**a**), charge capacity diagram of CNT forests by varying the ink concentration (**b**) and cyclic voltammograms of CNT forests by varying the ink concentration (**c**).

Catalyst morphology was further investigated since it might have significant role on the future properties of both individual carbon nanotubes and CNT forests. To characterize the catalyst layer blank CCVD experiments were carried out: every step was identical, only the carbon source (ethylene) was omitted from the system. In the absence of hydrocarbon obtaining catalyst layer close to pre-synthetic form was assumed. SEM images taken after blank experiments can be seen in Fig. 4a. Aluminum substrate is homogeneously covered by catalyst of various particle sizes in case of different ink concentrations, however, due to sintering their size cannot be determined precisely. While the depth of native oxide layer (after the treatment described in the previous section) was 10 nm, the average thickness of catalyst layer was found to be 20 nm.

From SEM analysis of both CNT forests and catalyst particles, it can be presumed that the density of CNT forest depends on the quality of catalyst layer deposited onto the surface of Al substrate. As it was already mentioned before (Fig. 3a) the density of CNT forest might depend on the ink concentration. To prove this assumption we attempted to measure the “concentration” of CNT forests. As a preliminary approach samples before and after CCVD synthesis (e.g. initial aluminum substrate with catalyst layer and the same with carbon deposit) were weighed. Since CNT forest contains more than 90% air, the accuracy of this method is only approximate. Nevertheless, from the increase in mass and from the height and area of CNT forest, density can be calculated. These approximate data can be found in Fig. 4b, and it can be concluded that the highest CNT forest can be obtained by applying catalyst ink of 0.11 M concentration.

In order to verify this result an independent technique, namely cyclic voltammetry was carried out on CNT forests prepared using different ink concentrations (Fig. 4c). From these results not only the density of the samples but the existence of electric contact between CNTs and Al support was also proved. From cyclic voltammograms charge capacity can be calculated directly, thus the electrochemically accessible surface can be determined. This parameter is in strong correlation with CNT forest density (assuming constant CNT diameter) since the sample contains more carbon nanotubes, which leads to higher surface area and higher density. This fact is of great importance because many potential applications (e.g. batteries, sensors, catalyst supports) are based on the available surface together with unique electric conductance of CNTs as well. Results confirmed the estimation of density obtained by the mass measurements.

### Changes of different parameters during CCVD synthesis

Adjusting the parameters of CCVD synthesis properly is very important for fine-tuning the structure of vertically aligned carbon nanotube arrays. In this section the effect of gas feed and reaction time will be discussed. In each case SEM analysis of carbon deposit was performed from which the height of CNT forests were determined.

#### The effect of gas feed

Firstly the effect of nitrogen, hydrogen and ethylene feed was investigated. While reaction time was fixed (15 mins), two parallel series were carried out: with and without water vapor. In order to investigate how nitrogen flow affects the synthesis, the gas feed was varied as follows: 50 *cm* ^3^ × *min* ^−1^, 60 *cm* ^3^ × *min* ^−1^, and 75 *cm* ^3^ × *min* ^−1^, respectively (increasing always by 20%), together with 70 *cm* ^3^ × *min* ^−1^ of ethylene and 100 *cm* ^3^ × *min* ^−1^ of hydrogen. Measurements were carried out either with or without water vapor. The composition of gas feed for different syntheses are summarized in Table 1.

**Table 1:** The composition of gas feed for different syntheses.

nitrogen	ethylene	hydrogen	water vapor
50	70	100	32
60			37
75			42
50	70	100	32
	95		37
	120		42
50	70	100	32
		110	37
		130	42
50	70	100	20
			30
			40
			50
			60

**Figure 5:**
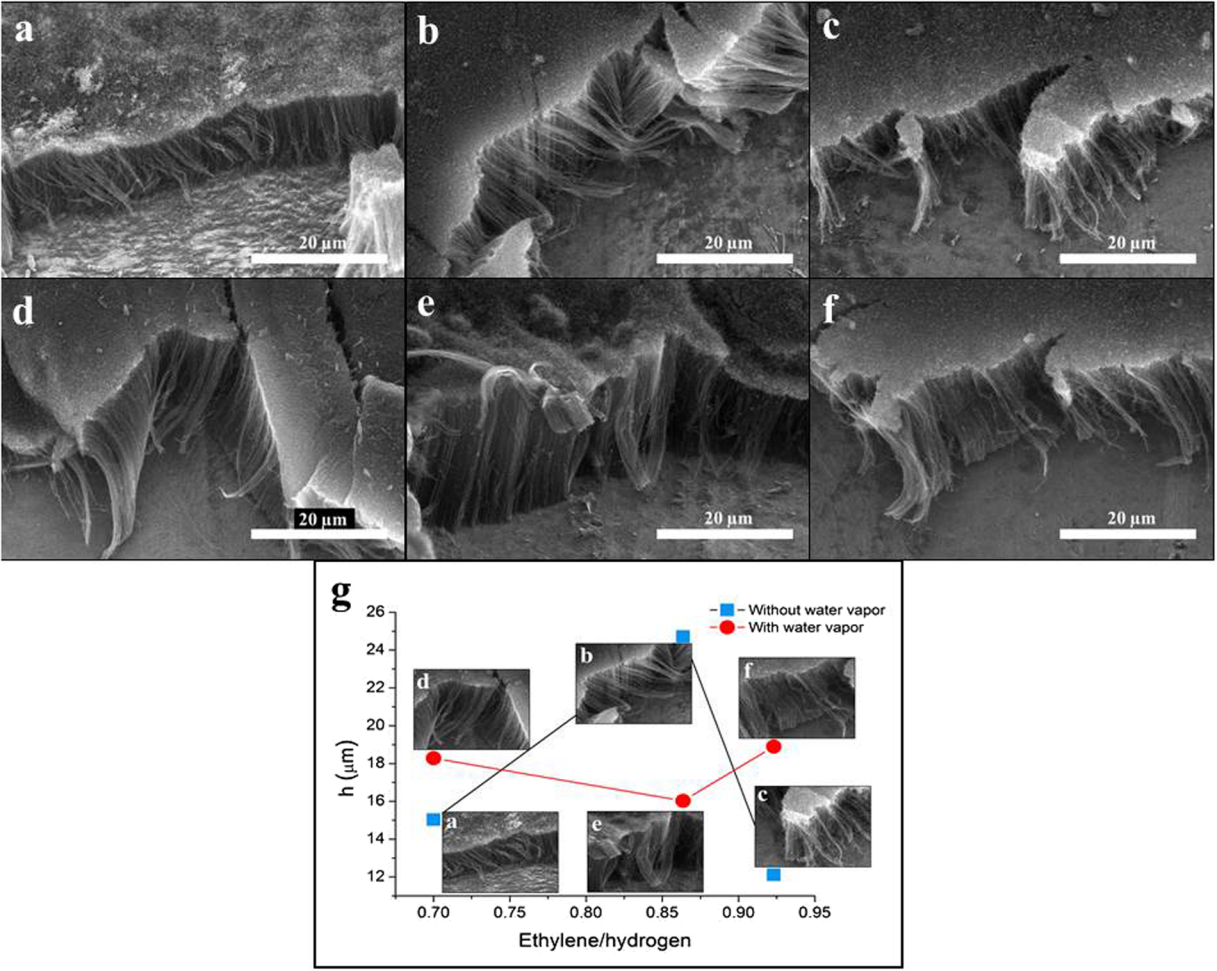
SEM images of CNT forests synthesized by varying the nitrogen gas feed without water vapor 50 *cm* ^3^ × *min* ^−1^ (**a**), 60 *cm* ^3^ × *min* ^−1^ (**b**), and 75 *cm* ^3^ × *min* ^−1^ (**c**), SEM images of CNT forests synthesized by varying the nitrogen gas feed with water vapor 50 *cm* ^3^ × *min* ^−1^ (**d**), 60 *cm* ^3^ × *min* ^−1^ (**e**), and 75 *cm* ^3^ × *min* ^−1^ (**f**), and heights of CNT forests (**g**).

From SEM analysis both the height and alignment of CNT forests were determined. Figure 5 shows SEM images of carbon nanotube forests synthesized by different nitrogen flow either in the presence ((d)-(f)) or the absence ((a)-(c)) of water vapor. It can be seen that the quality, the orientation of the samples are always better if the gas feed contained water. This feature can be attributed to the fact that without water vapor the system contains more defects, resulting in chaotic like orientation of the carbon nanotube. In the case when water vapor in present in the system it facilitates the inhibition of defects, also it can oxidize the present amorphous carbon in the system, supporting the desired orientation of the carbon nanotube forests. Concerning the quality similar observations were made when the flow of ethylene or hydrogen were altered.

Plotted heights of CNT forests fabricated under various conditions are summarized in Fig. 5g. From these data it can be concluded that water vapor has significant influence not only on the alignment of the samples but on their height too. While the highest CNT forest obtained was 24.7 µm using the lowest gas feed without water vapor (Fig. 5b), the lowest one was 12.1 µm using the highest gas feed without water vapor (Fig. 5c). It can be deduced that CNT forest’s height is decreasing with increasing gas feed, however, this tendency cannot be applied in the presence of water vapor.

#### The effect of water vapor feed

Since the effect of water on the CNT growth is indisputable its influence was investigated more thoroughly. Results pointed out that water assisted CCVD synthesis provides CNT forest of better quality, moreover, the presence of water affects the height as well. These two effects are probably due to the oxidizing feature of water vapor which facilitates the removal of less graphitized carbon nanotubes (thus providing better orientation) on one hand and controls/reduces its height on the other^10^. Therefore, the optimization of water vapor content in the gas feed is crucial. In these experiments the flow rate of nitrogen, ethylene and hydrogen was constant, 50 *cm* ^3^ × *min* ^−1^, 70 *cm* ^3^ × *min* ^−1^, and 100 *cm* ^3^ × *min* ^−1^, respectively, and the water vapor feed was varied in the range of 20 to 60 *cm* ^3^ × *min* ^−1^.

**Figure 6:**
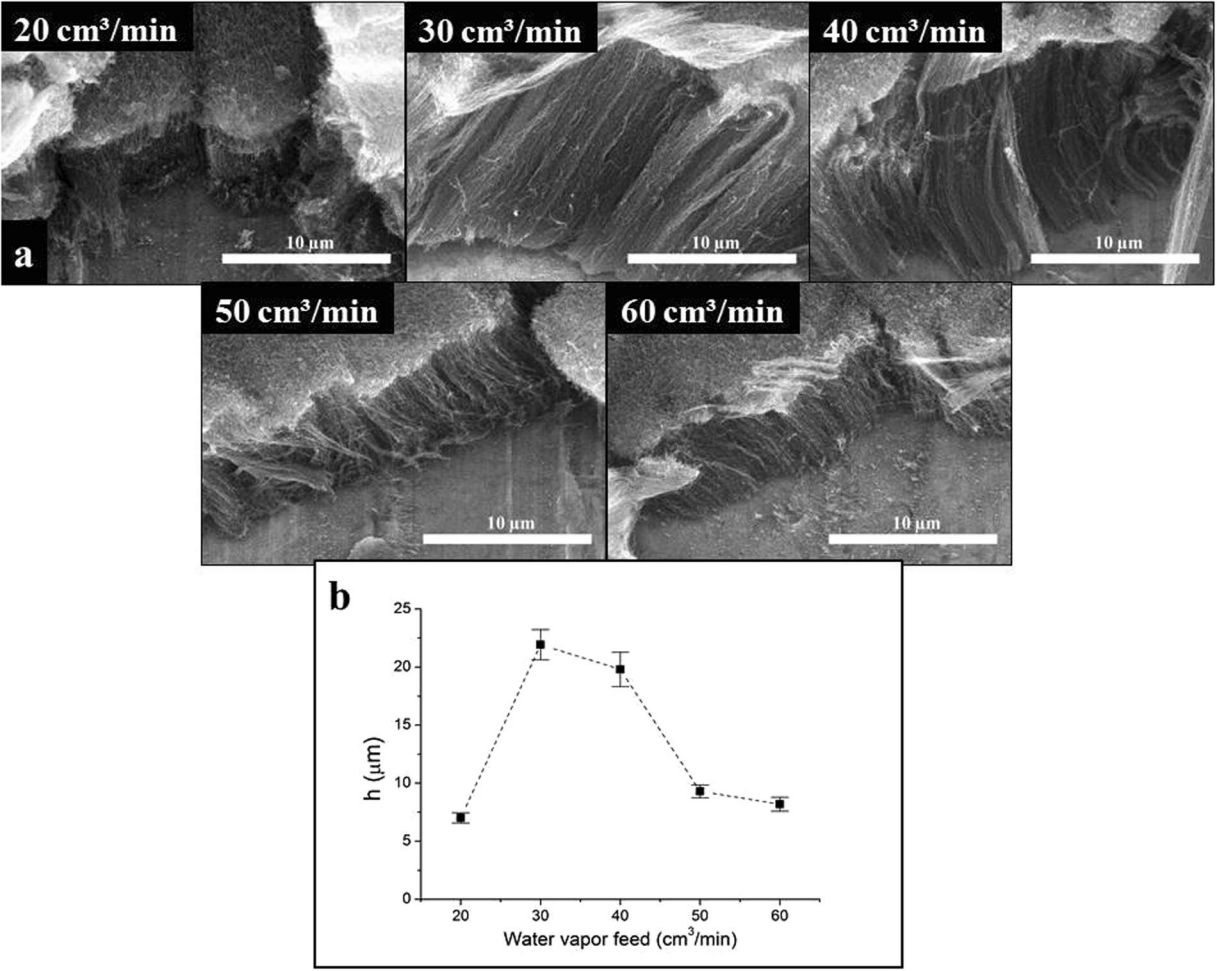
SEM images of CNT forests synthesized by varying the water vapor feed (**a**) and heights of CNT forests (**b**).

SEM investigation (Fig. 6a) revealed that the highest CNT forest (21.9 µm) was obtained in case of 30 *cm* ^3^ × *min* ^−1^ water vapor flow. If lower value is applied, the CNT forest is consisted of sinuous carbon nanotubes with the smallest observed height (7 µm). Although when the concentration of water vapor was increased it had a negative effect on the heights of the graphitic carbon nanotube forests (their height was reduced), which was discussed above. Increasing the water vapor feed (see Fig. 6b) resulted in the decrease of height of CNT forest which can be explained by the reaction of carbon nanotubes with water vapor. E.g. the rate of CNT growth becomes lower than the rate of decomposition (discussed before).

### The effect of reaction time

The effect of reaction time was investigated in the next series in which all gas flows were kept constant. In these experiments nitrogen was used to purge the system for 1.5 min then to reduce the catalyst hydrogen was added to the gas flow for 5 min. CVD reaction was started with launching the flow of both ethylene and water vapor. Considering the optimum water vapor feed described in the previous section it was worth to study at which reaction time the presence of water starts to hinder the growth of CNTs. Therefore the effect of reaction time was investigated in the range of 5 to 60 minutes.

**Figure 7:**
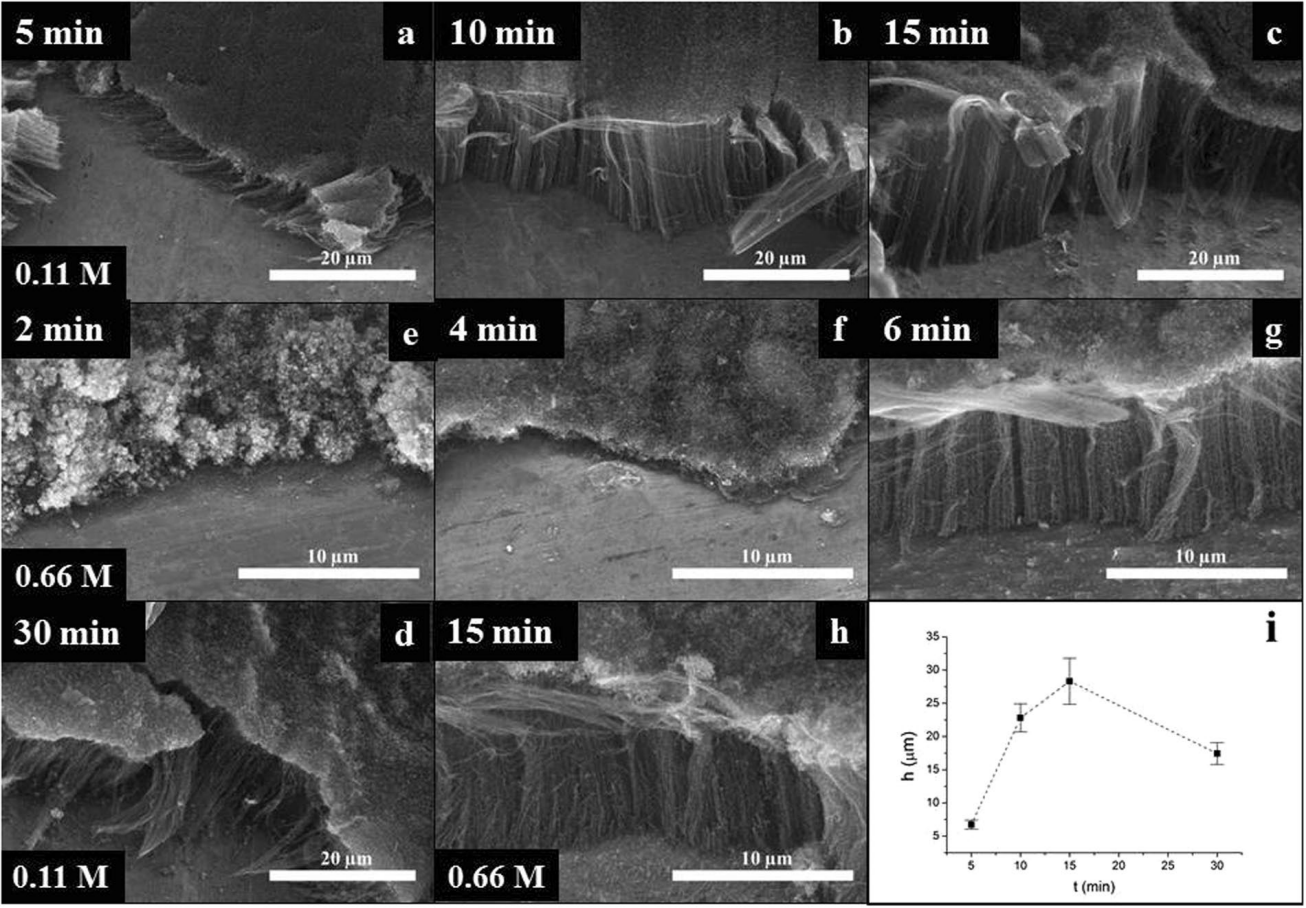
SEM images of CNT forests synthesized by varying the reaction time with 0.11 M ink concentration (**a–d**), SEM images of CNT forests synthesized by varying the reaction time with 0.66 M ink concentration (**e–h**) and heights of CNT forests with 0.11 M ink concentration (**i**).

It is well known from the literature that the alignment of CNT forests is mainly due to van der Waals interactions between growing nanotubes, and the steric hindrance between neighboring CNTs^39^. From SEM images (Fig. 7) it can be concluded that after 5 min the system reaches the stage when alignment has just started, and CNT forest of moderate height (6.7 µm) was observed. At the very beginning of the CVD synthesis CNTs are growing inordinately non-perpendicular to the substrate surface. At a certain length of the tubes – due to the above-mentioned interactions – their orientation is initiated resulting in “forest formation”. Reaction time longer than only 5 mins provided a much higher orderliness of the product, both the quality and the length of carbon nanotubes improved significantly (Fig. 7b). From Fig. 7i it can be seen that under these conditions the highest carbon nanotube forest of 28 µm can be obtained after 15 min. After that, probably due to the gradual inactivation of catalyst particles the growth of carbon nanotubes stalls, and the synthesis as well as the degradation by continuous water vapor feed becomes competitive resulting in the stagnation of height of the forest. With further deactivation of the catalyst, reaction with water vapor starts to dominate and in approximately 60 min the complete disappearance of the carbon deposit occurs.

It was already observed in the literature that internal constraints might cause vertical orientation during CVD that can be facilitated by increasing the catalyst layer on the surface of the substrate, resulting in a so-called “crowding effect”^40^. In order to determine if the synthesis time can be further reduced, catalyst ink of the highest concentration (0.66 M) was investigated at different reaction times in the range of 2 to 15 minutes. SEM measurements (Fig. 7) revealed the properties of carbon deposit after various CVD synthesis periods. Experiments at 2–4–6 minutes (Fig. 7e–g, respectively) thoroughly demonstrated the evolvement of CNT forest. After 2 minutes carbon deposit appeared, however, neither carbon nanotubes nor orderliness of forming fibers could be recognized. During the synthesis of 4 minutes the quality of carbon deposit has changed significantly, but no characteristic alignment could be observed. 6 minutes of reaction time was the shortest period when well-oriented CNT forest of 12.5 µm could be observed. In case of 15 min reaction time the height of CNT forest became 18.5 µm (Fig. 7h.) From this series it can be concluded that increasing the concentration of the catalyst ink the necessary reaction time can be somewhat reduced.

### Further analysis of carbon nanotubes composing forests

#### TEM

**Figure 8:**
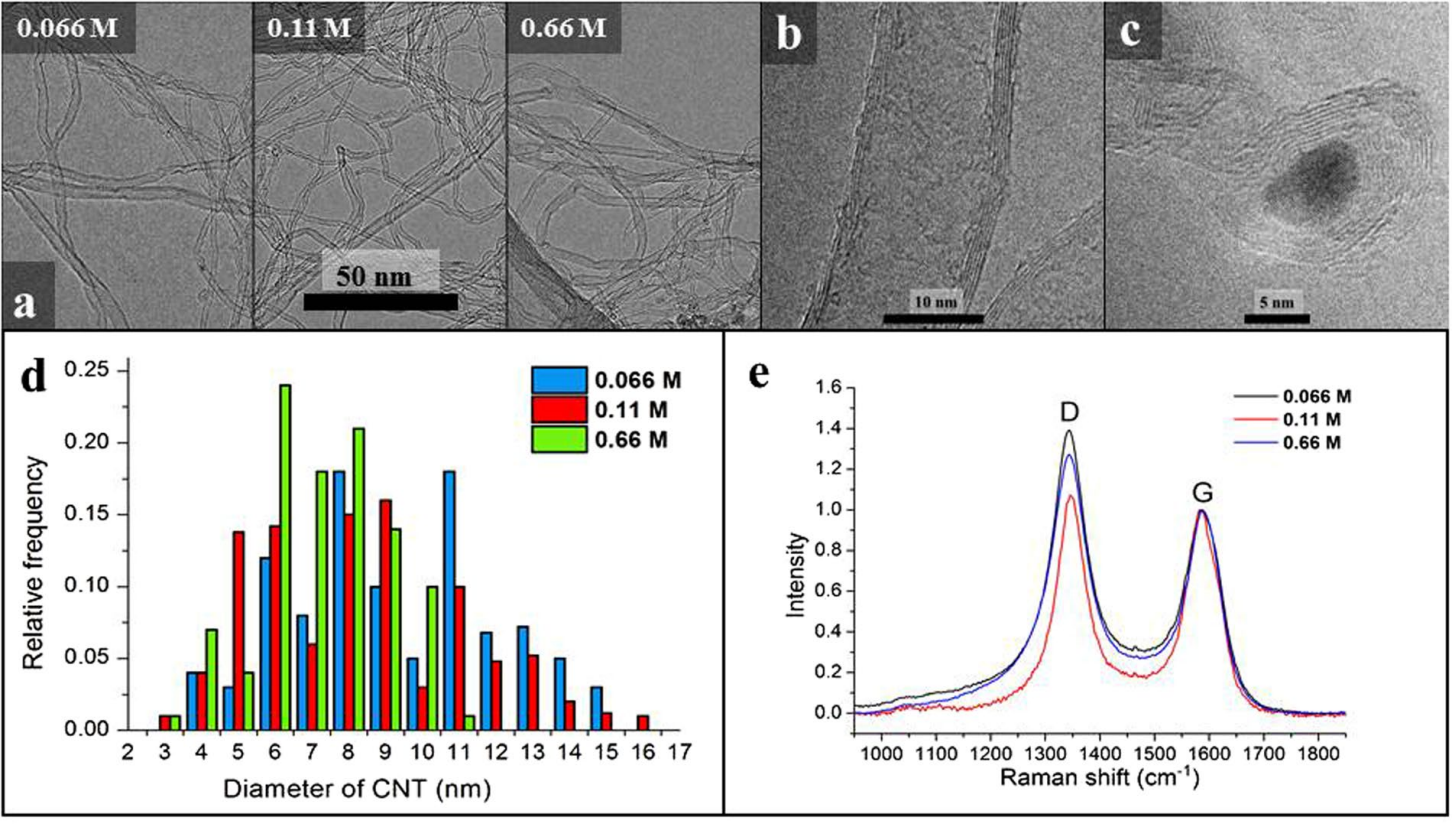
TEM images of CNT forests synthesized by varying the ink concentration (**a**), TEM images of the CNT walls (**b**), TEM images of the catalyst particle (**c**), the diameter distribution of CNT (**d**) and Raman spectra of the CNT forests of different ink concentration (**e**).

To determine the quality of carbon nanotubes composing the forest TEM analysis was also carried out (Fig. 8a). HR images verified the high-level graphitization of their walls which consist of 3 to 6 walls in average (Fig. 8b). These investigations also proved that these forests are grown by the root mechanism^41, 42^. However, in certain regions separated catalyst particles covered by thick graphite layer could be observed (Fig. 8c). As an illustration, carbon nanotubes prepared with catalyst ink concentration of 0.066 M, 0.11 M and 0.66 M, respectively, are shown in Fig. 8d. The outer diameter of the CNTs is in the range of 6 to 10 nm, and there is only an insignificant difference in these values as a function of concentration. From TEM images it can be also observed that the surfaces of CNTs are rather clean, i.e. outer surfaces contains almost no amorphous layers of carbon.

#### Raman spectroscopy

Figure 8e shows representative Raman spectra of CNT forest samples prepared by catalyst ink concentration of 0.066 M, 0.11 M and 0.66 M, respectively. Practically these spectra are identical which means that there is no significant difference between the qualities of carbon nanotubes. Their I_*G*_/I_*D*_ ratios are 1.07 +/−0.04. This result is in accordance with the observations from TEM investigations. From literature data it can be supposed that increasing CVD temperatures result in an even higher level of graphitization^43^, however, in our case aluminum substrate of relatively low melting point demands a temperature limit for the synthesis.

## Conclusion

In this study, a cheap and easy method was presented for the production of VACNTs onto conductive substrate. It was shown that CNT forest height grown onto an Al plate during CCVD synthesis can be controlled by numerous parameters. When varying catalyst ink concentration during the formation of catalyst layer via dip coating, the highest forest was obtained at Fe:Co = 1:3 ratio. Increasing the catalyst ink concentration, it was found that approximately above 0.11 M the height of the forest did not change significantly. The components of gas feed during CCVD also affected the parameters of forming CNT forests, however, water vapor has the most determinate role on their height. In accordance with former literature observations it was established that no considerable alignment occurs when CNTs are shorter than 10 µm. Above this value the orderliness of CNT forests becomes conspicuous more or less independently of either reaction time or catalyst ink concentration. However, the water vapor and reaction time influence both the carbon nanotube forests height and orientation. From the analysis of CNT diameter it was concluded that the catalyst ink concentration does not affect the diameter of catalyst particles significantly. It is presumed that both the quality of the ink and CCVD conditions control the diameter of CNT. Authors expect that this simple and cheap method will open up novel applications in nanotechnology devices.

## References

[1] Li WZ (1996). Large-scale synthesis of aligned carbon nanotubes. Sci..

[2] Noda S (2007). Millimeter-thick single-walled carbon nanotube forests: Hidden role of catalyst support. Jpn. J. Appl. Physics, Part 2: Lett..

[3] Mattevi C (2008). *In-situ* X-ray photoelectron spectroscopy study of catalyst- support interactions and growth of carbon nanotube forests. The J. Phys. Chem. C.

[4] Sakurai S (2011). Role of subsurface diffusion and ostwald ripening in catalyst formation for SWNT forest growth. J Am Chem Soc.

[5] Robertson J (2012). Applications of carbon nanotubes grown by chemical vapor deposition. Jpn. J. Appl. Phys..

[6] Halonen N (2008). Controlled CCVD synthesis of robust multiwalled carbon nanotube films. J. Phys. Chem. C.

[7] Hata K (2004). Water-assisted highly efficient synthesis of impurity-free single-walled carbon nanotubes. Sci..

[8] Jiang C, Liang Y, Yang J, Zhao B (2013). Influence of total gas flow on carbon nanotube forests synthesised by water-assisted chemical vapour deposition. Micro & Nano Lett..

[9] Mauron P, Emmenegger C, Zuttel A, Nutzenadel C, Schlapbach L (2002). Synthesis of oriented nanotube films by chemical vapor deposition. Carbon Nanotub..

[10] Futaba DN (2005). Kinetics of water-assisted single-walled carbon nanotube synthesis revealed by a time-evolution analysis. Phys. Rev. Lett..

[11] Tripathi N, Mishra P, Harsh H, Islam SS (2014). Fine-tuning control on CNT diameter distribution, length and density using thermal CVD growth at atmospheric pressure: an in-depth analysis on the role of flow rate and flow duration of acetylene (C2H2) gas. Appl. Nanosci..

[12] Patole S, Alegaonkar P, Lee H-C, Yoo J-B (2008). Optimization of water assisted chemical vapor deposition parameters for super growth of carbon nanotubes. Carbon.

[13] Tripathi N, Mishra P, Joshi B, Islam S (2015). Precise control over physical characteristics of carbon nanotubes by differential variation of argon flow rate during Chemical Vapor Deposition processing: A systematic study on growth kinetics. Mater. Sci. Semicond. Process..

[14] Ma Y, Dichiara AB, He D, Zimmer L, Bai J (2016). Control of product nature and morphology by adjusting the hydrogen content in a continuous chemical vapor deposition process for carbon nanotube synthesis. Carbon.

[15] D L Arcos T, Oelhafen P, Thommen V, Mathys D (2007). The influence of catalyst’s oxidation degree on carbon nanotube growth as a substrate-independent parameter. J. Phys. Chem. C.

[16] Burt DP (2009). Effects of metal underlayer grain size on carbon nanotube growth. J. Phys. Chem. C.

[17] Lacerda RG (2004). Thin-film metal catalyst for the production of multi-wall and single-wall carbon nanotubes. J. Appl. Phys..

[18] Ren AZF (1998). Synthesis of large arrays of well-aligned carbon nanotubes on glass. Sci..

[19] Yao Y, Falk LKL, Morjan RE, Nerushev OA, Campbell EEB (2004). Synthesis of carbon nanotube films by thermal CVD in the presence of supported catalyst particles. Part II: The nanotube film. J. Mater. Sci. Mater. Electron..

[20] Eres G, Puretzky AA, Geohegan DB, Cui H (2004). *In situ* control of the catalyst efficiency in chemical vapor deposition of vertically aligned carbon nanotubes on predeposited metal catalyst films. Appl. Phys. Lett..

[21] Teblum E, Gofer Y, Pint CL, Nessim GD (2012). Role of catalyst oxidation state in the growth of vertically aligned carbon nanotubes. J Phys Chem C.

[22] Dubosc M (2007). Impact of the Cu-based substrates and catalyst deposition techniques on carbon nanotube growth at low temperature by PECVD. Microelectron. Eng..

[23] Fejes D (2015). Super growth of vertically aligned carbon nanotubes on pulsed laser deposited catalytic thin films. Appl. Phys. A: Mater. Sci. Process..

[24] Murakami Y, Miyauchi Y, Chiashi S, Maruyama S (2003). Direct synthesis of high-quality single-walled carbon nanotubes on silicon and quartz substrates. Chem. Phys. Lett..

[25] Dürfler S (2012). Tailoring structural and electrochemical properties of vertical aligned carbon nanotubes on metal foil using scalable wet-chemical catalyst deposition. J. Power Sources.

[26] Li G, Chakrabarti S, Schulz M, Shanov V (2009). Growth of aligned multiwalled carbon nanotubes on bulk copper substrates by chemical vapor deposition. J. Mater. Res..

[27] Magrez A, Seo JW, Smajda R, Mionić M, Forró L (2010). Catalytic CVD synthesis of carbon nanotubes: Towards high yield and low temperature growth. Mater..

[28] D S Meneses D, Malki M, Echegut P (2006). Structure and lattice dynamics of binary lead silicate glasses investigated by infrared spectroscopy. J. Non-Crystalline Solids.

[29] Johnson PB, Christy RW (1975). Optical constants of copper and nickel as a function of temperature. Phys. Rev. B.

[30] Lichtenstein, T. Handbook of Thin Film Materials. *Coll. Eng. Appl. Sci. Univ. Rochester* (1979).

[31] Dijon J (2010). How to switch from a tip to base growth mechanism in carbon nanotube growth by catalytic chemical vapour deposition. Carbon.

[32] Mata D (2012). Upscaling potential of the CVD stacking growth method to produce dimensionally-controlled and catalyst-free multi-walled carbon nanotubes. Carbon.

[33] Dresselhaus MS, Dresselhaus G, Saito R, Jorio A (2005). Raman spectroscopy of carbon nanotubes. Phys. Reports.

[34] Antunes EF (2006). Comparative study of first- and second-order Raman spectra of MWCNT at visible and infrared laser excitation. Carbon.

[35] Kind H (2000). Printing gel-like catalysts for the directed growth of multiwall carbon nanotubes. Langmuir.

[36] Smajda R (2009). Synthesis and mechanical properties of carbon nanotubes produced by the water assisted CVD process. Phys. Status Solidi (B) Basic Res..

[37] Kaneko A, Yamada K, Kumahara R, Kato H, Homma Y (2012). Comparative Study of Catalytic Activity of Iron and Cobalt for Growing Carbon Nanotubes on Alumina and Silicon Oxide. The J. Phys. Chem..

[38] Magrez A (2011). Striking influence of the catalyst support and its acid-base properties: New insight into the growth mechanism of carbon nanotubes. ACS Nano.

[39] Bower C, Zhu W, Jin S, Zhou O (2000). Plasma-induced alignment of carbon nanotubes. Appl. Phys. Lett..

[40] Nozaki T, Ohnishi K, Okazaki K, Kortshagen U (2007). Fabrication of vertically aligned single-walled carbon nanotubes in atmospheric pressure non-thermal plasma CVD. Carbon.

[41] Yang J (2015). Growth of high-density carbon nanotube forests on conductive TiSiN supports. The J. Phys. Chem..

[42] Sugime H (2013). Low temperature growth of ultra-high mass density carbon nanotube forests on conductive supports. Appl. Phys. Lett..

[43] Andrews R, Jacques D, Qian D, Rantell T (2002). Multiwall Carbon Nanotubes: Synthesis and Application. Acc. Chem. Res..

